# Supplementary material for: Elucidating the role of metal ions in carbonic anhydrase catalysis
Source: Nat Commun. 2020 Sep 11;11:4557. doi: 10.1038/s41467-020-18425-5 (PMC7486293; doi:10.1038/s41467-020-18425-5)
Supplement: Supplementary file 1 — Supplementary Information [file 41467_2020_18425_MOESM1_ESM.pdf]

Supplementary Information

Elucidating the role of metal ions in carbonic anhydrase catalysis

J. K. Kim *et al*.

This PDF file includes:

Supplementary Figure 1 - HCO_3^- binding geometry in Zn-, Co-, Ni-CA II.

Supplementary Figure 2 - Substrate binding in Zn-CA II at pH 7.8 and 11.0.

Supplementary Figure 3 - Product binding in Ni-CA II at pH 7.8 and 11.0.

Supplementary Figure 4 - Absorbance of Apo- & Zn-CA II in esterase kinetic assay at pH 7.8

Supplementary Figure 5 - Partial occupancy determination of His64 in CA II structures.

Supplementary Table 1 - Data collection and refinement statistics for the CA II structures.

Supplementary Table 2 - List of key bound water molecules in the CA II structures.

Supplementary Table 3 - Distance geometry in the CA II structures.

Supplementary References

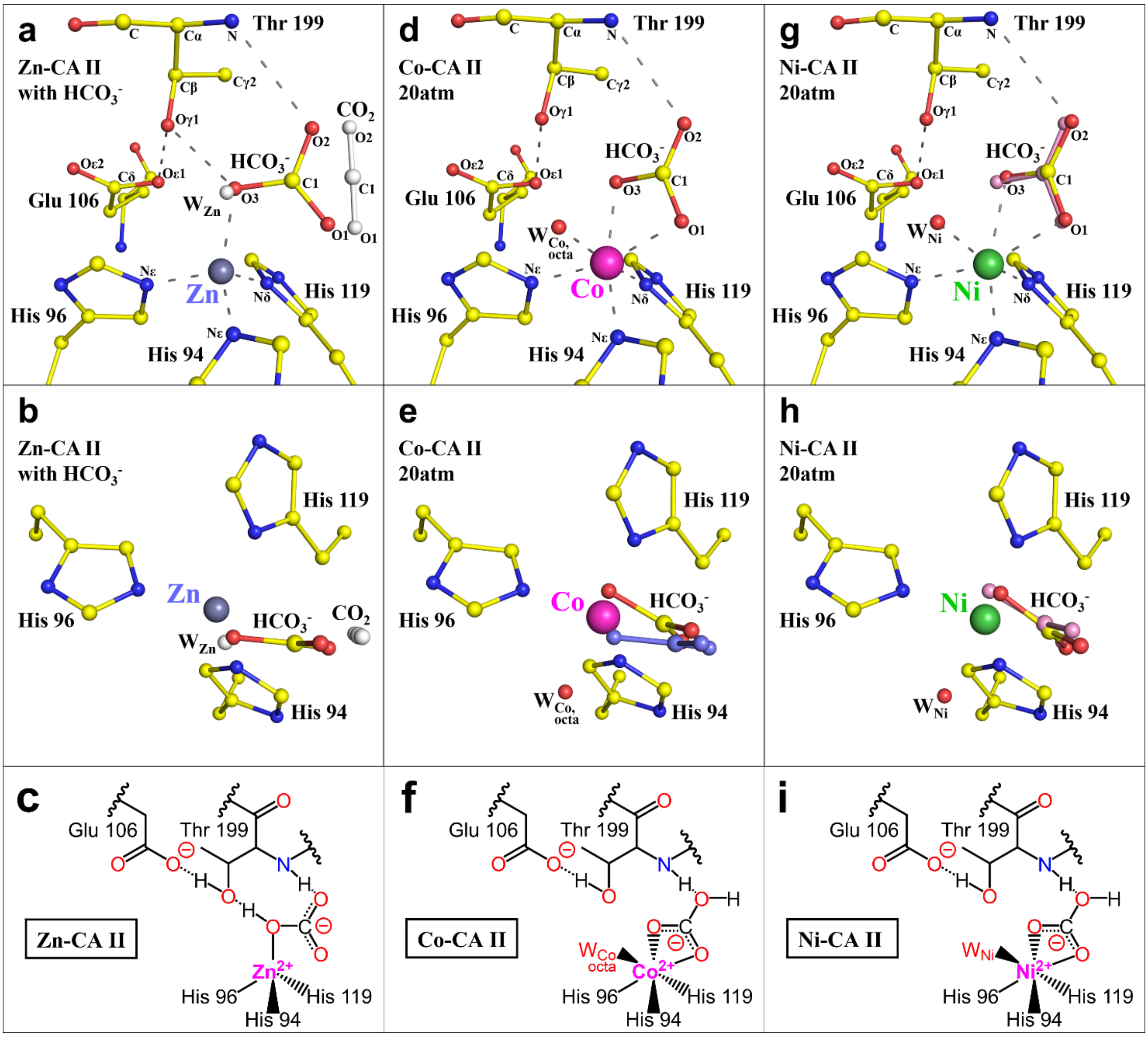

Supplementary Fig. 1. HCO_3^- binding geometry in Zn-, Co-, Ni-CA II. a-c) Monodentate binding of HCO_3^- in Zn-CA II (PDB code: 2vvb)[1]. The CO_2 molecule and W_{Zn} (white) from Zn-CA II 20atm are superimposed for comparison. Note that HCO_3^- lies on the plane made by the CO_2 molecule and W_{Zn}. **d-f**) Bidentate binding of HCO_3^- in Co-CA II 20atm pH 7.8. The HCO_3^- molecule from Zn-CA II (light purple, PDB 2vvb) is superimposed for comparison. Note that the HCO_3^- molecule in Co-CA II is tilted by ~ 31° to the HCO_3^- molecule in Zn-CA II. **g-i**) Bidentate binding of HCO_3^- in Ni-CA II 20atm. The HCO_3^- molecule in Co-CA II (pink) is superimposed for comparison.

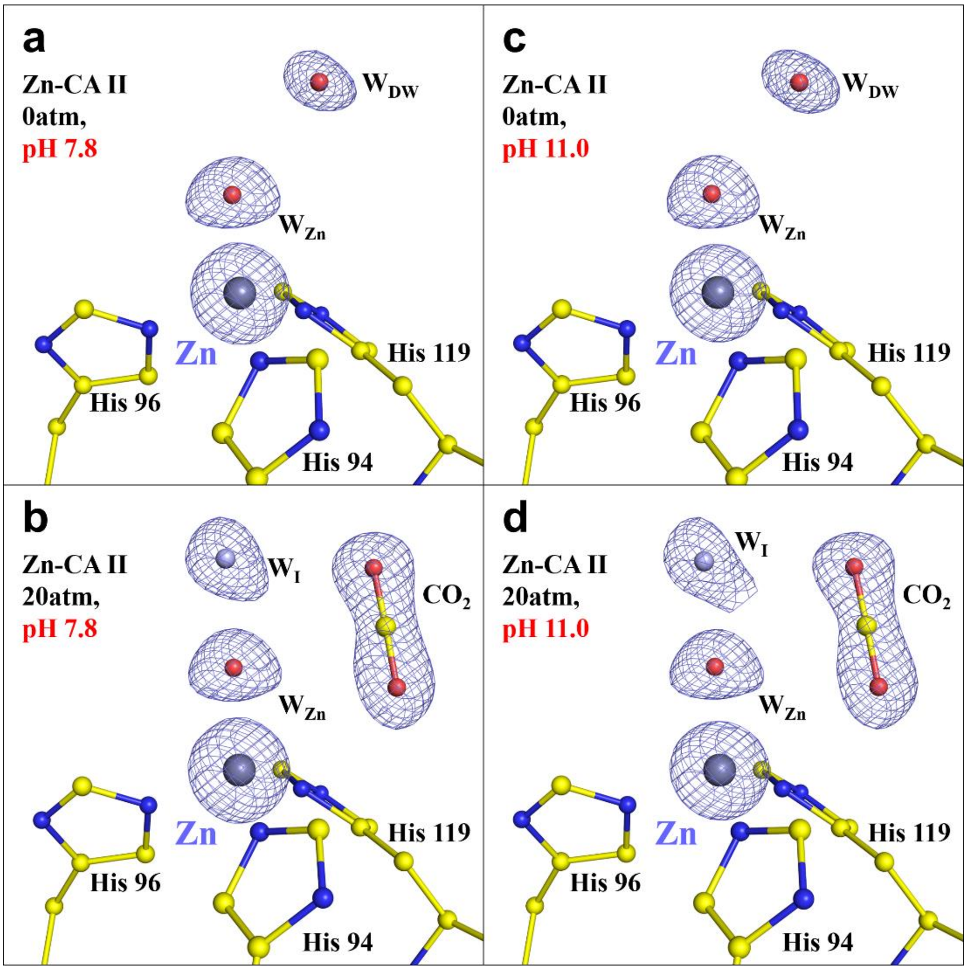

Supplementary Fig. 2. Substrate binding in Zn-CA II at pH 7.8 (a-b) and 11.0 (c-d). The metal coordination maintains tetrahedral geometry upon CO_2 binding regardless of pH values. The electron density ($2F_o$-F_c, blue) is contoured at 2.2σ. The intermediate water (W_I) is colored in steel blue for clarity.

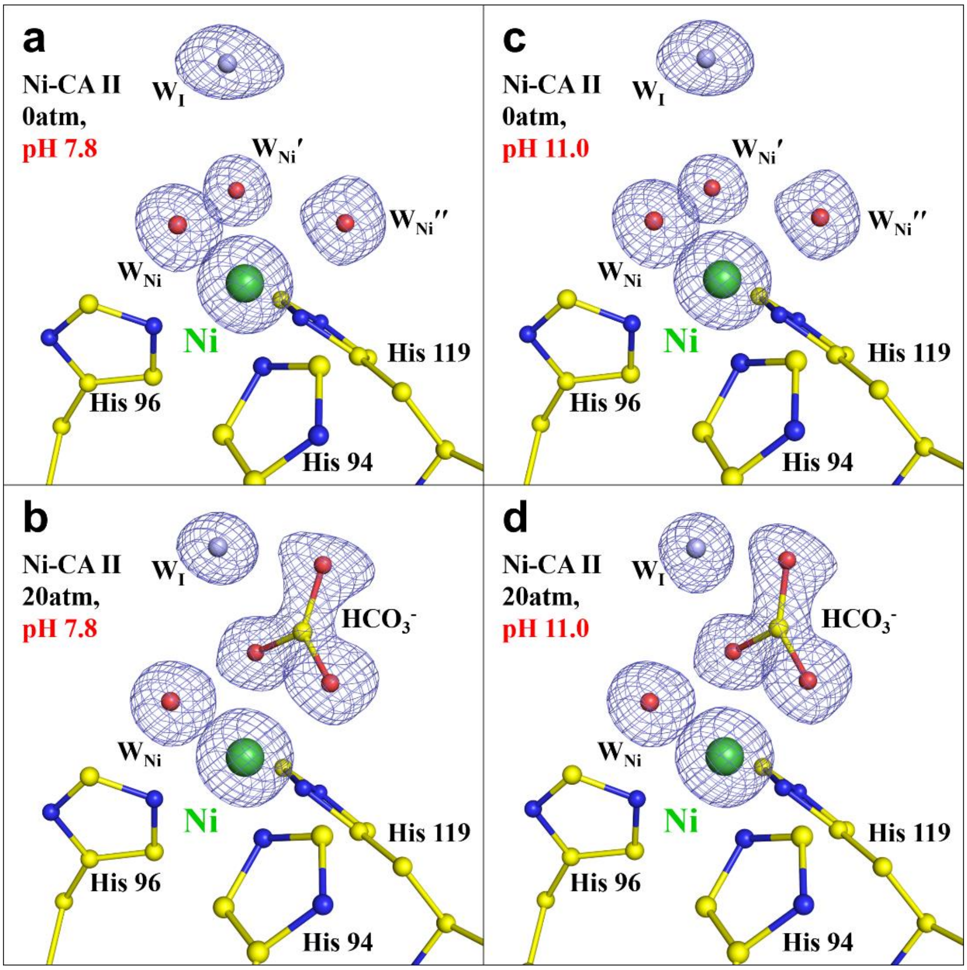

Supplementary Fig. 3. Product binding in Ni-CA II at pH 7.8 (a-b) and 11.0 (c-d). The metal coordination maintains octahedral geometry upon HCO_3^- binding regardless of pH values. The intermediate water (W_I) is colored in steel blue for clarity. The electron density ($2F_o$-F_c, blue) is contoured at 2.2σ.

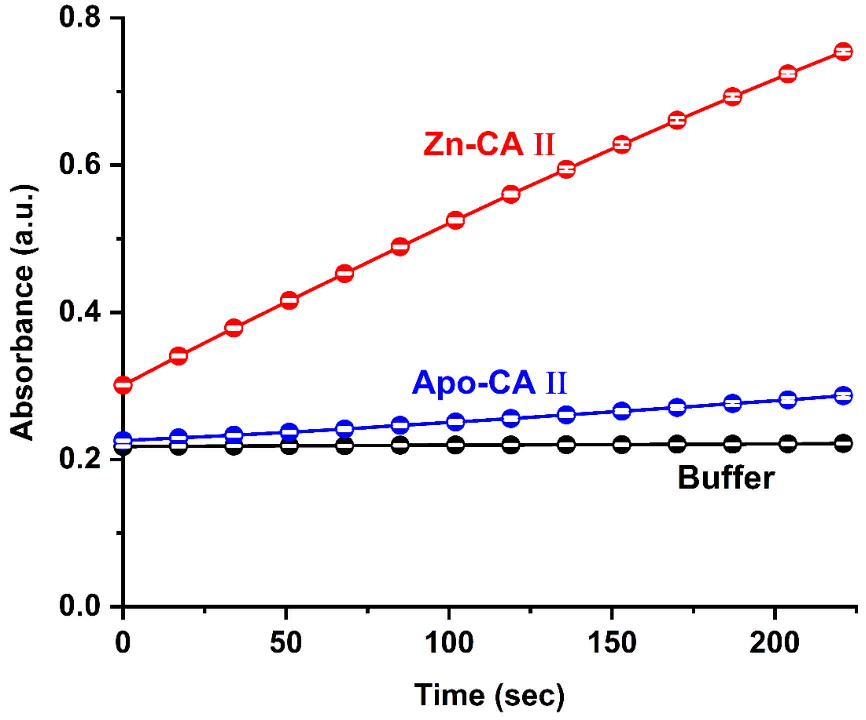

Supplementary Fig. 4. Absorbance of Apo- and Zn-CA II in esterase kinetic assay at pH 7.8. CA II esterase activity was measured spectroscopically at 348nm, indicative of substrate 4-nitrophenyl acetate hydrolysis. Compared to Zn-CA II, Apo-CA II and buffer show little to no esterase activity. The standard deviation errors (white) are presented in the data points and are ranging from 0.2 % ~ 1.1 %. Source data are provided as a Source Data file.

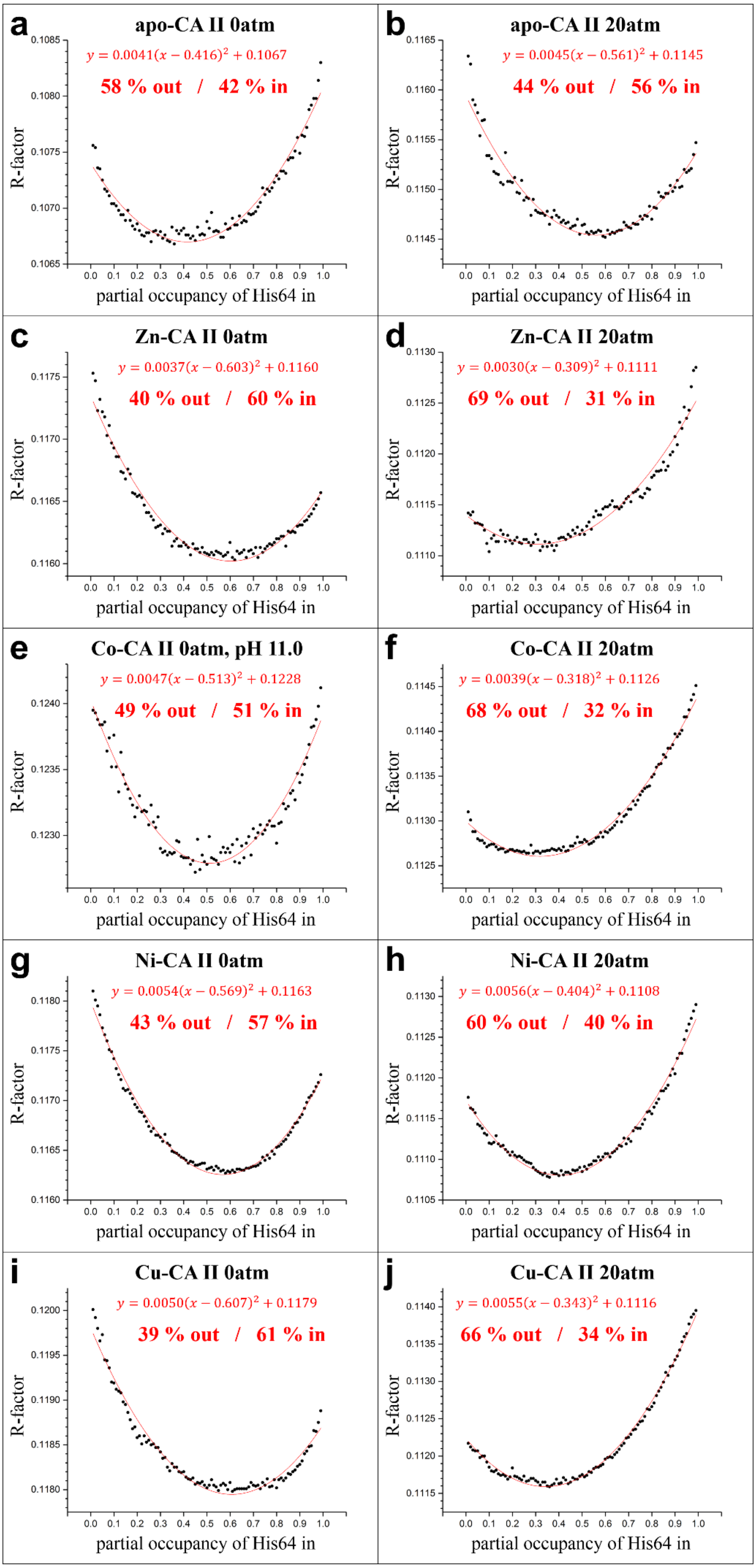

Supplementary Fig. 5. See next page for figure caption.

Supplementary Fig. 5. Partial occupancy determination of His64. The His64 out/in conformations are determined for the selected structures of apo-CA II (**a, b**) Zn-CA II (**c, d**), Co-CA II (**e, f**), Ni-CA II (**g, h**) Cu-CA II (**i, j**). For each data set, systematic refinements were carried out on 99 structures with manually adjusted His64 in/out occupancies. The obtained data points were then fitted to quadratic functions, showing the minimum points in the overall R factors. The His64 out/in conformations for the other structures can be found in Supplementary Table 1. Source data are provided as a Source Data file.

Supplementary Table 1. Data collection and refinement statistics for the CA II structures.

	apo-CA II<br>0atm<br><br>6LUU	apo-CA II<br>20atm<br><br>6LUV	Zn-CA II<br>0atm<br><br>6LUW	Zn-CA II<br>20atm<br><br>6LUX	Zn-CA II<br>0atm<br>pH11.0<br>6LUY	Zn-CA II<br>20atm<br>pH11.0<br>6LUZ	Co-CA II<br>0atm<br><br>6LV1	Co-CA II<br>20atm<br><br>6LV2
**Data collection**								
Space group	$P2_1$	$P2_1$	$P2_1$	$P2_1$	$P2_1$	$P2_1$	$P2_1$	$P2_1$
Cell dimensions								
a, *b*, *c* (Å)	42.13,<br>41.30,<br>72.21	42.26,<br>41.38,<br>72.00	42.21,<br>41.28,<br>72.15	42.37,<br>41.44,<br>72.13	42.28,<br>41.26,<br>72.07	42.39,<br>41.47,<br>72.15	42.31,<br>41.22,<br>72.05	42.32,<br>41.32,<br>72.22
β(°)	104.27	104.18	104.18	104.05	104.19	104.11	104.16	104.03
Resolution (Å)	30-1.20<br>(1.22-1.20)	30-1.20<br>(1.22-1.20)	30-1.20<br>(1.22-1.20)	30-1.20<br>(1.22-1.20)	30-1.20<br>(1.22-1.20)	30-1.20<br>(1.22-1.20)	30-1.20<br>(1.22-1.20)	30-1.20<br>(1.22-1.20)
R_{sym} (%)	5.8 (36.8)	7.1 (48.1)	6.5 (37.6)	6.8 (66.9)	8.5 (17.7)	5.6 (20.3)	9.2 (60.7)	8.2 (65.6)
$I/\sigma(I)$	28.8 (6.8)	21.2 (5.1)	29.7 (6.3)	29.8 (3.6)	25.2 (12.7)	29.9 (11.8)	24.3 (3.2)	20.1 (3.8)
Completeness (%)	95.5 (92.6)	94.3 (91.2)	98.8 (97.5)	96.2 (92.9)	98.0 (96.0)	94.6 (91.9)	96.0 (93.3)	98.4 (96.4)
Redundancy	7.5 (7.3)	7.5 (7.4)	7.3 (7.1)	7.4 (7.3)	7.4 (7.4)	7.6 (7.5)	7.6 (7.6)	7.4 (7.2)
**Refinement**								
Resolution (Å)	1.20	1.20	1.20	1.20	1.20	1.20	1.20	1.20
No. reflections	72,011	71,305	74,561	73,183	73,898	72,112	72,478	74,640
R_{work} / R_{free} (%)	11.0 / 13.8	11.7 / 14.8	11.7 / 14.1	11.3 / 14.2	11.1 / 13.1	10.3 / 12.7	12.0 / 15.0	11.4 / 13.9
No. atoms								
Protein	4,285	4,256	4,253	4,245	4,253	4,245	4,259	4,240
Ligand/ion	1 glycerol	2 CO_2,<br>1 glycerol	1 glycerol	2 CO_2,<br>1 glycerol	1 glycerol	2 CO_2,<br>1 glycerol	1 HCO_3^-,<br>1 glycerol	1 HCO_3^-,<br>1 CO_2,<br>1 glycerol
Water	371	373	263	367	264	365	283	333
B-factors								
Protein<br>(main / side chain)	9.78 / 12.75	11.48 / 14.38	10.09 / 13.22	10.80 / 13.62	7.90 / 10.99	8.87 / 11.51	10.84 / 14.07	10.31 / 13.31
Ligand/ion	20.76<br>(glycerol)	13.90<br>(first CO_2),<br>26.78<br>(second CO_2),<br>18.39<br>(glycerol)	18.32<br>(glycerol)	10.63<br>(first CO_2),<br>23.50<br>(second CO_2),<br>17.17<br>(glycerol)	14.79<br>(glycerol)	8.80<br>(first CO_2),<br>21.13<br>(second CO_2),<br>14.78<br>(glycerol)	11.23<br>(HCO_3^-),<br>24.46<br>(glycerol)	9.25<br>(HCO_3^-),<br>23.17<br>(second CO_2),<br>21.27<br>(glycerol)
Water	30.25	30.67	26.12	30.58	24.33	27.75	27.41	29.71
R.m.s. deviations								
Bond lengths (Å)	0.031	0.030	0.029	0.028	0.029	0.028	0.031	0.028
Bond angles (°)	2.515	2.435	2.437	2.423	2.506	2.368	2.644	2.284
Partial occupancy (%)								
His64 (out/in) conformation (%)	58 / 42	44 / 56	40 / 60	69 / 31	32 / 68	63 / 37	49 / 51	68 / 32

	Co-CA II<br>0atm<br>pH11.0<br>6LV3	Co-CA II<br>20atm<br>pH11.0<br>6LV4	Ni-CA II<br>0atm<br><br>6LV5	Ni-CA II<br>20atm<br><br>6LV6	Ni-CA II<br>0atm<br>pH11.0<br>6LV7	Ni-CA II<br>20atm<br>pH11.0<br>6LV8	Cu-CA II<br>0atm<br><br>6LV9	Cu-CA II<br>20atm<br><br>6LVA
**Data collection**								
Space group	$P2_1$	$P2_1$	$P2_1$	$P2_1$	$P2_1$	$P2_1$	$P2_1$	$P2_1$
Cell dimensions								
a, *b*, *c* (Å)	42.33,<br>41.26,<br>72.08	42.36,<br>41.46,<br>72.31	42.40,<br>41.29,<br>71.92	42.40,<br>41.37,<br>72.13	42.52,<br>41.23,<br>71.87	42.42,<br>41.40,<br>72.19	42.33,<br>41.23,<br>72.09	42.36,<br>41.40,<br>72.31
β(°)	104.13	104.02	104.03	104.01	104.06	104.04	104.17	103.97
Resolution (Å)	30-1.20<br>(1.22-1.20)	30-1.20<br>(1.22-1.20)	30-1.20<br>(1.22-1.20)	30-1.20<br>(1.22-1.20)	30-1.20<br>(1.22-1.20)	30-1.20<br>(1.22-1.20)	30-1.20<br>(1.22-1.20)	30-1.20<br>(1.22-1.20)
R_{sym} (%)	8.5 (46.4)	5.8 (42.4)	8.6 (33.1)	7.2 (37.7)	8.5 (29.7)	4.7 (22.7)	9.9 (42.8)	7.8 (44.8)
$I/\sigma(I)$	23.0 (4.3)	31.8 (5.6)	20.7 (7.6)	23.1 (7.2)	23.1 (8.4)	40.6 (10.1)	19.5 (6.0)	25.3 (5.5)
Completeness (%)	95.0 (91.7)	94.6 (91.2)	96.6 (93.8)	95.4 (92.1)	97.5 (94.7)	96.1 (93.0)	95.1 (92.3)	96.9 (94.3)
Redundancy	7.6 (7.7)	7.6 (7.6)	7.5 (7.4)	7.6 (7.5)	7.4 (7.3)	7.5 (7.3)	7.7 (7.6)	7.5 (7.5)

**Refinement**								
Resolution (Å)	1.20	1.20	1.20	1.20	1.20	1.20	1.20	1.20
No. reflections	71,822	72,158	73,007	72,507	73,791	73,217	71,843	73,841
R_{work} / R_{free} (%)	12.3 / 14.9	11.3 / 13.9	11.6 / 14.1	11.2 / 13.8	11.7 / 13.9	11.2 / 13.6	11.7 / 14.4	11.3 / 14.2
No. atoms								
Protein	4,252	4,240	4,259	4,240	4,259	4,240	4,259	4,240
Ligand/ion	1 glycerol	1 HCO_3^-, 2 CO_2, 1 glycerol	1 glycerol	1 HCO_3^-, 1 CO_2, 1 glycerol	1 glycerol	1 HCO_3^-, 1 CO_2, 1 glycerol	1 glycerol	1 CO_2, 1 glycerol
Water	265	333	279	347	279	346	286	360
B-factors								
Protein (main / side chain)	10.84 / 14.06	10.39 / 13.16	8.73 / 11.74	7.93 / 10.50	9.15 / 12.17	8.38 / 10.83	9.71 / 12.55	8.23 / 10.89
Ligand/ion	21.51 (glycerol)	15.78 (HCO_3^-), 14.19 (first CO_2), 21.78 (second CO_2), 19.39 (glycerol)	18.86 (glycerol)	9.15 (HCO_3^-), 21.92 (second CO_2), 14.87 (glycerol)	19.13 (glycerol)	7.40 (HCO_3^-), 21.95 (second CO_2), 13.93 (glycerol)	17.19 (glycerol)	19.67 (second CO_2), 14.81 (glycerol)
Water	26.98	28.72	24.56	26.58	26.51	26.38	26.62	29.36
R.m.s. deviations								
Bond lengths (Å)	0.031	0.028	0.032	0.029	0.030	0.028	0.031	0.029
Bond angles (°)	2.484	2.358	2.600	2.382	2.552	0.352	2.550	2.460
Partial occupancy (%)								
His64 (out/in) conformation (%)	49 / 51	69 / 31	43 / 57	60 / 40	46 / 54	52 / 48	39 / 61	66 / 34

*Values in parentheses are for the highest-resolution shell.

Supplementary Table 2. List of key bound water molecules in the CA II structures.

	apo-CA II 0atm 6LUU	apo-CA II 20atm 6LUV	Zn-CA II 0atm 6LUW	Zn-CA II 20atm 6LUX	Co-CA II 0atm pH11.0 6LV3	Co-CA II 20atm 6LV2	Ni-CA II 0atm 6LV5	Ni-CA II 20atm 6LV6	Cu-CA II 0atm 6LV9	Cu-CA II 20atm 6LVA
CO_2	–	A 302		A 302	–		–		–	–
HCO_3^-	–	–			–	A 304	–	A 305	–	–
W_{Zn}	–	–	A 481	A 426	–		–	–	–	–
$W_{Co, tetra}$	–	–	–	–	A 490		–	–	–	–
$W_{Co, octa}$	–	–	–	–	–	A 485	–	–	–	–
W_{Ni}	–	–	–	–	–	–	A 573	A 583	–	–
W_{Ni}'	–	–	–	–	–	–	A 421	–	–	–
W_{Ni}''	–	–	–	–	–	–	A 602	–	–	–
W_{Cu}	–	–	–	–	–	–	–	–	A 451	A 421
W_{Cu}'	–	–	–	–	–	–	–	–	A 580	–
W_{apo}	A 486	A 475		–	–	–	–	–	–	–
W_{DW}	A 618	–	A 581	–	A 569	–	–	–	A 587	A 496
W_{DW}'	–	–	–	–	–	–	–	–	–	A 695
W_{DW}''	–	–	–	–	–	–	–	–	–	A 675
W_I	–	A 591	–	A 483	–	A 448	A 611	A 495	–	A 427
W_I'	–	–	–	A 643	–	–	–	–	–	A 663
W1	A 591	–	A 487		A 546	–	–	–	A 563	
W2	A 641	A 666	A 615	A 678	A 584	A 667	A 627	A 662	A 636	A 681
W2′	–	–	–	A 401	–	A 401	–	–	–	A 401
W3a	A 541	A 556	A 518	A 543	A 529	A 537	A 532	A 507	A 540	A 528
W3b	A 462	A 430	A 451	A 451	A 454	A 432	A 453	A 479	A 442	A 452
W3b′	–	–	–	A 695	–	–	–	–	–	A 684
$W3b_{apo}'$	–	A 606	–	–	–	–	–	–	–	–
$W3b_{apo}'$	–	A 703	–	–	–	–	–	–	–	–
$W3b_{Co}$	–	–	–	–	–	A 648	–	–	–	–
W_{EC1}	A 680	–	A 614	A 665	A 611	A 630	–	–	A 619	A 648
W_{EC1}'	–	–	–	A 702	–	–	–	–	–	A 699
W_{EC1}''	–	–	–	–	–	–	A 598	A 626	–	
W_{EC2}	A 668	A 700	A 608	–	A 609	–	–	–	A 603	A 641
W_{EC2}'	–	–	–	A 697	–	A 661	A 649	A 669	–	A 697
W_{EC2}''	–	–	–	–	–	–	A 613	A 642	–	–
W_{EC2}'''	–	–	–	–	–	A 673	–	–	–	–
W_{EC3}	A 707	A 712	A 634	A 720	A 639	A 706	A 658	A 704	A 649	A 703
W_{EC3}'	–	A 714	–	–	–	–	–	–	–	–
W_{EC3}''	–	A 710	–	–	–	–	–	–	–	–
W_{EC4}	A 761	A 760	A 655	A 755	A 660	A 720	A 672	A 734	A 676	
W_{EC5}	A 447	A 421	A 438	A 417	A 437	A 430	A 442	A 428	A 420	A 502

Supplementary Table 3. Distance geometry (Å) of CO_2, HCO_3^- and key bound water molecules in the CA II structures.

	apo-CA II 0atm 6LUU	apo-CA II 20atm 6LUV	Zn-CA II 0atm 6LUW	Zn-CA II 20atm 6LUX	Co-CA II 0atm pH11.0 6LV3	Co-CA II 20atm 6LV2	Ni-CA II 0atm 6LV5	Ni-CA II 20atm 6LV6	Cu-CA II 0atm 6LV9	Cu-CA II 20atm 6LVA
Zn – W_{Zn}	–	–	1.88	1.92	–	–	–	–	–	–
Zn – CO_2(O1)	–	–	–	3.31	–	–	–	–	–	–
Co – $W_{Co,\ tetra}$	–	–	–	–	1.72	–	–	–	–	–
Co – $W_{Co,\ octa}$	–	–	–	–	–	2.03	–	–	–	–
Co – CO_2(O1)	–	–	–	–	–	–	–	–	–	–
Co – HCO_3^-(O1)	–	–	–	–	–	2.07	–	–	–	–
Co – HCO_3^-(O3)	–	–	–	–	–	2.02	–	–	–	–
Ni – W_{Ni}	–	–	–	–	–	–	2.09	2.13	–	–
Ni – W_{Ni}'	–	–	–	–	–	–	2.14	–	–	–
Ni – W_{Ni}''	–	–	–	–	–	–	2.22	–	–	–
Ni – HCO_3^-(O1)	–	–	–	–	–	–	–	2.16	–	–
Ni – HCO_3^-(O3)	–	–	–	–	–	–	–	2.18	–	–
Cu – W_{Cu}	–	–	–	–	–	–	–	–	2.17	2.26
Cu – W_{Cu}'	–	–	–	–	–	–	–	–	2.36	–
Cu – W_{DW}''	–	–	–	–	–	–	–	–	–	3.14
CO_2(C1) – W_{Zn}	–	–	–	2.87	–	–	–	–	–	–
CO_2(C1) – $W_{Co,\ tetra}$	–	–	–	–	–	–	–	–	–	–
HCO_3^-(O1) – $W_{Co,\ octa}$	–	–	–	–	–	2.85	–	–	–	–
HCO_3^-(O3) – $W_{Co,\ octa}$	–	–	–	–	–	2.72	–	–	–	–
HCO_3^-(O1) – W_{Ni}	–	–	–	–	–	–	–	2.92	–	–
HCO_3^-(O3) – W_{Ni}	–	–	–	–	–	–	–	2.85	–	–
CO_2(C1) – W_{apo}	–	2.94	–	–	–	–	–	–	–	–
W_{Zn} – W_{DW}	–	–	2.58	–	–	–	–	–	–	–
W_{Zn} – W1	–	–	2.63	–	–	–	–	–	–	–
W_{Zn} – W_I	–	–	–	2.75	–	–	–	–	–	–
W_{Zn} – W2	–	–	4.41	4.65	–	–	–	–	–	–
$W_{Co,\ tetra}$ – W_{DW}	–	–	–	–	2.61	–	–	–	–	–
$W_{Co,\ tetra}$ – W1	–	–	–	–	2.73	–	–	–	–	–
$W_{Co,\ tetra}$ – W_I	–	–	–	–	–	–	–	–	–	–
$W_{Co,\ tetra}$ – W2	–	–	–	–	4.55	–	–	–	–	–
$W_{Co,\ octa}$ – W_I	–	–	–	–	–	2.75	–	–	–	–
$W_{Co,\ octa}$ – W2	–	–	–	–	–	2.71	–	–	–	–
$W_{Co,\ octa}$ – W2′	–	–	–	–	–	4.90	–	–	–	–
W_{Ni} – W_{Ni}'	–	–	–	–	–	–	2.82	–	–	–
W_{Ni} – W_{Ni}''	–	–	–	–	–	–	3.08	–	–	–
W_{Ni}' – W_{Ni}''	–	–	–	–	–	–	2.78	–	–	–
W_{Ni} – W_I	–	–	–	–	–	–	2.95	2.88	–	–
W_{Ni} – W2	–	–	–	–	–	–	2.73	2.68	–	–
W_{Cu} – W1	–	–	–	–	–	–	–	–	2.54	–
W_{Cu} – W_{Cu}'	–	–	–	–	–	–	–	–	3.06	–

W_{Cu} – W_{DW}	–	–	–	–	–	–	–	–	3.23	2.84
W_{Cu}' – W_{DW}	–	–	–	–	–	–	–	–	2.84	–
W_{DW} – W_{DW}'	–	–	–	–	–	–	–	–	–	1.52
W_{DW}' – W_{DW}''	–	–	–	–	–	–	–	–	–	1.60
W_{Cu} – W_{I}	–	–	–	–	–	–	–	–	–	2.84
W_{Cu} – W2	–	–	–	–	–	–	–	–	3.44	3.90
W_{apo} – W_{DW}	2.71	–	–	–	–	–	–	–	–	–
W_{apo} – W1	2.82	–	–	–	–	–	–	–	–	–
W_{apo} – W_{I}	–	2.88	–	–	–	–	–	–	–	–
W_{apo} – W2	4.90	3.96	–	–	–	–	–	–	–	–
W1 – W2	2.73	–	2.74	–	2.70	–	–	–	2.78	–
W_{I} – W_{I}'	–	–	–	2.02	–	–	–	–	–	2.09
W2 – W2′	–	–	–	1.43	–	2.79	–	–	–	1.51
W2 – W3a	2.75	2.80	2.77	3.12	2.77	3.40	2.81	2.72	2.81	3.14
W2 – W3b	2.72	2.99	2.73	2.49	2.68	2.41	2.95	3.08	2.79	2.57
W2 – W3b′	–	–	–	1.68	–	2.69	–	–	–	1.65
W3b – W3b′	–	–	–	1.37	–	1.86	–	–	–	1.39
Thr199(N) – HCO_3^-(O2)	–	–	–	–	–	2.93	–	3.05	–	–
Thr199(N) – CO_2(O2)	–	3.20	–	3.62	–	–	–	–	–	–
Thr199(N) – W_{DW}	2.93	–	2.92	–	2.90	–	–	–	2.90	2.74
Thr199(N) – W_{I}	–	3.34	–	3.52	–	3.71	3.04	3.52	–	3.68
Thr199(Oγ1) – HCO_3^-(O3)	–	–	–	–	–	2.44	–	2.46	–	–
Thr199(Oγ1) – W_{Zn}	–	–	2.73	2.62	–	–	–	–	–	–
Thr199(Oγ1) – $W_{Co, tetra}$	–	–	–	–	2.75	–	–	–	–	–
Thr199(Oγ1) – $W_{Co, octa}$	–	–	–	–	–	3.75	–	–	–	–
Thr199(Oγ1) – W_{Ni}	–	–	–	–	–	–	3.68	3.65	–	–
Thr199(Oγ1) – W_{Ni}'	–	–	–	–	–	–	2.62	–	–	–
Thr199(Oγ1) – W_{Ni}''	–	–	–	–	–	–	5.04	–	–	–
Thr199(Oγ1) – W_{Cu}	–	–	–	–	–	–	–	–	2.68	2.61
Thr199(Oγ1) – W_{Cu}'	–	–	–	–	–	–	–	–	4.37	–
Thr199(Oγ1) – W_{apo}	2.72	2.69	–	–	–	–	–	–	–	–
Thr199(Oγ1) – Glu106(Oε1)	2.60	2.59	2.61	2.56	2.62	2.53	2.91	2.55	2.61	2.59
Glu106(Oε1) – W_{Zn}	–	–	4.15	4.01	–	–	–	–	–	–
Glu106(Oε1) – $W_{Co, tetra}$	–	–	–	–	4.08	–	–	–	–	–
Glu106(Oε1) – $W_{Co, octa}$	–	–	–	–	–	4.85	–	–	–	–
Glu106(Oε1) – W_{Ni}	–	–	–	–	–	–	4.75	4.84	–	–
Glu106(Oε1) – W_{Ni}'	–	–	–	–	–	–	2.78	–	–	–
Glu106(Oε1) – W_{Cu}	–	–	–	–	–	–	–	–	4.20	4.19

Glu106(Oε1) – W_{apo}	4.01	4.09	–	–	–	–	–	–	–	–
His64$_{in}$(Nδ1) – W2	3.02	3.16	3.22	3.28	2.94	3.88	3.29	3.22	3.43	3.48
His64$_{in}$(Nδ1) – W2′	–	–	–	1.86	–	1.26	–	–	–	1.99

Supplementary References

1 Sjoeblom, B., Polentarutti, M. & Djinovic-Carugo, K. Structural study of X-ray induced activation of carbonic anhydrase. *Proc Natl Acad Sci U S A* **106**, 10609-10613, doi:10.1073/pnas.0904184106 (2009).
