# Supplementary material for: Elucidating the role of metal ions in carbonic anhydrase catalysis
Source: Nat Commun. 2020 Sep 11;11:4557. doi: 10.1038/s41467-020-18425-5 (PMC7486293; doi:10.1038/s41467-020-18425-5)
Supplement: Supplementary file 3 — reporting summary [file 41467_2020_18425_MOESM3_ESM.pdf]

nature research

Corresponding author(s): Chae Un Kim
Last updated by author(s): Aug 16, 2020

Reporting Summary

Nature Research wishes to improve the reproducibility of the work that we publish. This form provides structure for consistency and transparency in reporting. For further information on Nature Research policies, see our Editorial Policies and the Editorial Policy Checklist.

Statistics

For all statistical analyses, confirm that the following items are present in the figure legend, table legend, main text, or Methods section.

n/a	Confirmed	
x	☐	The exact sample size (*n*) for each experimental group/condition, given as a discrete number and unit of measurement
x	☐	A statement on whether measurements were taken from distinct samples or whether the same sample was measured repeatedly
x	☐	The statistical test(s) used AND whether they are one- or two-sided<br>*Only common tests should be described solely by name; describe more complex techniques in the Methods section.*
x	☐	A description of all covariates tested
x	☐	A description of any assumptions or corrections, such as tests of normality and adjustment for multiple comparisons
x	☐	A full description of the statistical parameters including central tendency (e.g. means) or other basic estimates (e.g. regression coefficient) AND variation (e.g. standard deviation) or associated estimates of uncertainty (e.g. confidence intervals)
x	☐	For null hypothesis testing, the test statistic (e.g. *F*, *t*, *r*) with confidence intervals, effect sizes, degrees of freedom and *P* value noted<br>*Give P values as exact values whenever suitable.*
x	☐	For Bayesian analysis, information on the choice of priors and Markov chain Monte Carlo settings
x	☐	For hierarchical and complex designs, identification of the appropriate level for tests and full reporting of outcomes
x	☐	Estimates of effect sizes (e.g. Cohen's *d*, Pearson's *r*), indicating how they were calculated

Our web collection on statistics for biologists contains articles on many of the points above.

Software and code

Policy information about availability of computer code

Data collection	The X-ray crystallographic data were collected at Pohang Light Source II (PLS2) in South Korea.<br>The structural biology beamlines at PLS2 have their own software to control their X-ray beamlines and to help data collection, just as all other synchrotron sources have customized software for their own purposes.<br>The beamline software programs are freely available to the beamline users.
Data analysis	We used HKL2000 (version v716.1) , CCP4 program suite (version 6.5.012), Refmac5 (version 5.8.0124), and COOT (version 0.8.1) for data analysis. These are well-known and some of the most widely used software in the structural biology community. No other customized software was used. We described the information in the "Methods" section.

For manuscripts utilizing custom algorithms or software that are central to the research but not yet described in published literature, software must be made available to editors and reviewers. We strongly encourage code deposition in a community repository (e.g. GitHub). See the Nature Research guidelines for submitting code & software for further information.

Data

Policy information about availability of data

All manuscripts must include a data availability statement. This statement should provide the following information, where applicable:

- Accession codes, unique identifiers, or web links for publicly available datasets
- A list of figures that have associated raw data
- A description of any restrictions on data availability

The atomic coordinates and structure factors have been deposited in the Protein Data Bank (http://wwpdb.org/) as [PDB code 6LUU [http://dx.doi.org/10.2210/pdb6luu/pdb] (0 atm CO2 pressure, pH 7.8), 6LUV [http://dx.doi.org/10.2210/pdb6luv/pdb] (20 atm, pH 7.8)] for apo-CA II, [6LUW [http://dx.doi.org/10.2210/pdb6luw/pdb] (0 atm, pH 7.8), 6LUX [http://dx.doi.org/10.2210/pdb6lux/pdb] (20 atm, pH 7.8), 6LUY [http://dx.doi.org/10.2210/pdb6luy/pdb] (0 atm, pH 11.0),

6LUZ [http://dx.doi.org/10.2210/pdb6luz/pdb] (20 atm, pH 11.0)] for Zn-CA II, [6LV1 [http://dx.doi.org/10.2210/pdb6lv1/pdb] (0 atm, pH 7.8), 6LV2 [http://dx.doi.org/10.2210/pdb6lv2/pdb] (20 atm, pH 7.8), 6LV3 [http://dx.doi.org/10.2210/pdb6lv3/pdb] (0 atm, pH 11.0), 6LV4 [http://dx.doi.org/10.2210/pdb6lv4/pdb] (20 atm, pH 11.0)] for Co-CA II, [6LV5 [http://dx.doi.org/10.2210/pdb6lv5/pdb] (0 atm, pH 7.8), 6LV6 [http://dx.doi.org/10.2210/pdb6lv6/pdb] (20 atm, pH 7.8), 6LV7 [http://dx.doi.org/10.2210/pdb6lv7/pdb] (0 atm, pH 11.0), 6LV8 [http://dx.doi.org/10.2210/pdb6lv8/pdb] (20 atm, pH 11.0)] for Ni-CA II, and [6LV9 [http://dx.doi.org/10.2210/pdb6lv9/pdb] (0 atm, pH 7.8), 6LVA [http://dx.doi.org/10.2210/pdb6lva/pdb] (20 atm, pH 7.8)] for Cu-CA II. Two earlier structures [5DSR [http://dx.doi.org/10.2210/pdb5dsr/pdb] and 5YUK [http://dx.doi.org/10.2210/pdb5yuk/pdb]] were used for structure determination. Source data are provided with this paper.

Field-specific reporting

Please select the one below that is the best fit for your research. If you are not sure, read the appropriate sections before making your selection.

- [x] Life sciences
- [ ] Behavioural & social sciences
- [ ] Ecological, evolutionary & environmental sciences

For a reference copy of the document with all sections, see nature.com/documents/nr-reporting-summary-flat.pdf

Life sciences study design

All studies must disclose on these points even when the disclosure is negative.

Sample size	For each CA II crystallographic structure, a total of 360 diffraction pattern images were obtained. Details on the data statistics can be found in the Supplementary information.
Data exclusions	All collected diffraction patterns were used for structural analysis.
Replication	All reported protein structures were deposited in Protein Data Bank (PDB) with internal statistical validation reports, ensuring the reliability of the data analysis.
Randomization	Randomization of data is not applicable to structure determination through X ray crystallography. Details on the data statistics are provided in the Supplementary Information.
Blinding	Structural determination was carried out following standard practice. Blinding is not applicable as no group allocation was involved.

Reporting for specific materials, systems and methods

We require information from authors about some types of materials, experimental systems and methods used in many studies. Here, indicate whether each material, system or method listed is relevant to your study. If you are not sure if a list item applies to your research, read the appropriate section before selecting a response.

Materials & experimental systems

n/a	Involved in the study
[x]	[ ] Antibodies
[x]	[ ] Eukaryotic cell lines
[x]	[ ] Palaeontology and archaeology
[x]	[ ] Animals and other organisms
[x]	[ ] Human research participants
[x]	[ ] Clinical data
[x]	[ ] Dual use research of concern

Methods

n/a	Involved in the study
[x]	[ ] ChIP-seq
[x]	[ ] Flow cytometry
[x]	[ ] MRI-based neuroimaging
